# Mandibular Prognathism in Dolang Sheep: Hi-C Evidence for Localized TAD Remodeling at Craniofacial Loci

**DOI:** 10.3390/ani16010039

**Published:** 2025-12-23

**Authors:** Chao Fang, Hang Cao, Lingling Liu, Wujun Liu

**Affiliations:** 1 College of Animal Sciences, Xinjiang Agricultural University, Urumqi 830052, China; 2 Faculte de Medecine Veterinaire, Universite de Liege, Quartier Vallee 2, Avenue de Cureghem 6 (B43), 4000 Liege, Belgium

**Keywords:** dolang sheep, mandibular prognathism, craniofacial development, 3D genome architecture, Hi-C, topologically associating domains

## Abstract

Mandibular prognathism (Class III malocclusion) is a craniofacial anomaly characterized by an anteriorly positioned mandible, a concave facial profile and impaired mastication, and appears unusually frequently in Dolang sheep (*Ovis aries*). We combined clinical phenotyping and three-dimensional (3D) genome profiling to investigate this trait in a Dolang sheep flock. We examined 959 animals using standardized criteria, estimated a local prevalence of 10.3%, and assembled a 200 affected/200 unaffected case–control cohort for genomic analyses. As an exploratory pilot study of 3D genome architecture, we generated in situ Hi-C datasets from mandibular bone of two affected and two control sheep. At 40 kb resolution, global topologically associating domain (TAD) organization and boundary strength were broadly conserved between groups, but sliding-window analyses identified a small number of 1 Mb hotspots where affected animals showed increased TAD-boundary density and strengthened insulation. These UNDER-enriched windows lay near genes with plausible roles in craniofacial development, including *ROBO2, COL27A1, VRK2* and a cytokine cluster (*IL22/IL26/IFNG* with *MDM1*). Together, our data indicate that mandibular prognathism in Dolang sheep is associated with localized remodeling of chromatin insulation at a restricted set of gene-proximal loci and highlight candidate regions and mechanisms for integration with whole-genome sequencing, association and transcriptomic data.

## Simple Summary

Dolang sheep in Xinjiang, China, show a relatively high frequency of mandibular prognathism (“reverse overbite”), which makes chewing difficult and may reduce welfare and productivity. In a field survey of 959 Dolang sheep from a commercial flock, we found that 10.3% were affected. Affected animals had a concave facial profile, a forward-positioned lower jaw and the lower incisors biting in front of the upper incisors. Based on this survey, we built a case–control resource (200 affected and 200 unaffected animals) and used radiographs and bone histology to describe the craniofacial changes. To explore possible genetic mechanisms, we also studied how DNA is folded inside the cell nucleus. Using a three-dimensional genome method (Hi-C) on mandibular bone from two affected and two control sheep, we found that most large-scale folding patterns were similar between groups. However, affected sheep showed small but clear local changes in genome folding next to genes involved in jaw and facial development. These findings suggest that reverse overbite in Dolang sheep may be linked to subtle changes in genome architecture at key craniofacial genes and support Dolang sheep as a useful large-animal model for human mandibular prognathism.

## 1. Introduction

Mandibular prognathism (Class III malocclusion) is a craniofacial anomaly characterized by an anteriorly positioned and/or elongated mandible, a concave facial profile, malocclusion and impaired mastication [1,2]. Beyond aesthetic concerns, the condition compromises oral function, feed intake and overall health, and in severe cases can lead to weight loss and reduced productivity [3]. In humans, mandibular prognathism and related Class III malocclusions show moderate to high heritability, and both familial aggregation and genome-wide studies point to a polygenic architecture with contributions from genes involved in cartilage, bone growth and craniofacial patterning [4].

Large domestic animals, particularly sheep, can serve as phenotypically relevant models for human craniofacial disorders because they share comparable skull size, bone biology and life-history traits, and allow practical, well-controlled sampling in agricultural settings. Mandibular prognathism and related jaw defects have been reported in several livestock species, including sheep, goats, cattle and horses, where they can impair grazing efficiency and lead to culling [1,2,4]. However, the genetic and regulatory mechanisms underlying these conditions in livestock remain poorly characterized compared with humans, and very few studies have examined genome-scale regulatory architecture in affected animals [5,6].

Dolang sheep are a meat–fat multipurpose breed in Xinjiang, China, prized for rapid growth, aseasonal estrus and high fertility [7,8]. In parts of Makit (Maigaiti) County, breeders have noted an elevated frequency of mandibular prognathism, yet systematic prevalence estimates and mechanistic insights have been lacking. In a regional screen of a commercial Dolang flock, we observed a prevalence of 10.3% for mandibular prognathism based on standardized extraoral and intraoral criteria, whereas other surveyed breeds did not show obvious cases. Because masticatory dysfunction can impair digestion, promote emaciation and ultimately reduce wool and meat yield, elucidating the biological basis of this defect is both economically and biologically important [9,10].

A variety of experimental strategies are available to visualize or infer three-dimensional (3D) genome organization. Microscopy-based approaches such as DNA FISH and 3D-FISH can directly image spatial distances between selected loci, but they are generally low-throughput and limited to a small number of targets per experiment. Chromosome conformation capture (3C) and its derivatives (4C, 5C and promoter/capture Hi-C) enrich for specific viewpoints or promoter–enhancer contacts and are well suited for fine-mapping known loci, whereas ChIA-PET and HiChIP tether chromatin interactions to selected DNA-binding proteins [11,12,13]. More recently, Micro-C has provided nucleosome-scale contact maps in selected systems, but it is technically demanding and not yet routine in large-animal tissues [14]. In contrast, genome-wide Hi-C and its in situ implementation provide an unbiased survey of chromatin contacts across the entire genome, enabling robust identification of A/B compartments, topologically associating domains (TADs) and insulation boundaries in a single experiment [12,15]. For the present study, we therefore adopted a standard in situ Hi-C protocol in mandibular bone, as it offers a practical balance between genome-wide coverage, resolution at the TAD/boundary scale and compatibility with crosslinked ovine tissue, and it has an extensive track record in mammalian 3D genome studies [14,15].

While DNA sequence variation underlies many inherited traits, higher-order genome organization also shapes craniofacial phenotypes by constraining or permitting enhancer–promoter communication across TADs and their boundaries [11,12]. Hi-C profiling of 3D genome folding therefore offers a complementary view to detect localized changes in chromatin insulation that may accompany mandibular abnormalities [12]. Integrating 3D genome information with population-based and transcriptomic evidence increases the chance of pinpointing mechanisms rather than correlations alone [13,16].

Here, we first establish standardized clinical criteria and a prevalence estimate for mandibular prognathism in Dolang sheep, and we present radiographic and histopathological characterization of affected versus unaffected animals. We then implement an exploratory, pilot in situ Hi-C study of mandibular bone in a subset of two affected and two control sheep, embedded within a larger case–control resource (200 cases and 200 controls) established for whole-genome and transcriptomic analyses. By analyzing TAD and boundary architecture, we aim to identify focal remodeling of chromatin insulation at craniofacial loci and to generate mechanistic hypotheses and candidate regions for subsequent integration with whole-genome sequencing, genome-wide association and RNA-seq data.

## 2. Materials and Methods

### 2.1. Ethical Approval

All procedures involving animals were reviewed and approved by the Animal Welfare and Ethics Committee of Xinjiang Agricultural University, Urumqi, China (Approval No. 2021103; approved on 30 October 2021). Sampling complied with national and local regulations and adhered to the ethical standards of the Animals journal. Reporting follows ARRIVE recommendations where applicable. Written permission for flock access and sampling was obtained from farm owners/managers.

### 2.2. Animals, Breed, Housing, and Grouping

All animals were Dolang sheep (*Ovis aries*) sourced from commercial farms in Makit (Maigaiti) County, Xinjiang, China. Sheep were maintained under standard husbandry conditions (group housing, routine forage–concentrate diet and ad libitum access to water). Basic management practices (deworming, vaccination and routine health checks) followed farm protocols. On the sampling day, general health status and fitness for sampling were confirmed by a licensed veterinarian.

**Figure 1:**
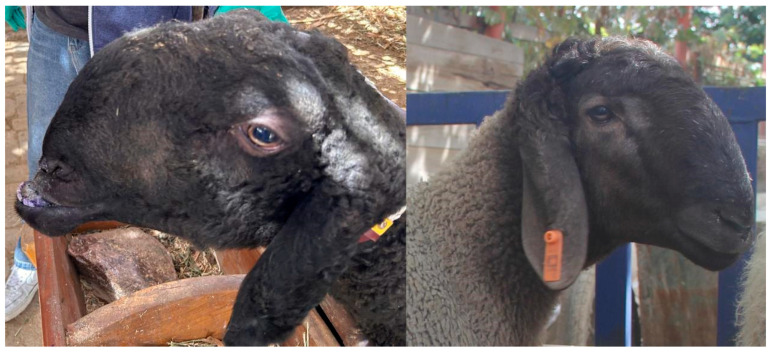
Representative facial phenotypes of Dolang sheep. (**Left**) A normal individual; (**right**) an individual exhibiting the underbite (mandibular prognathism) phenotype. All photographs were taken during this study from the same animals used for sampling and sequencing. Photos were taken by H.C. (Hang Cao), an author of this manuscript.

Phenotyping and case definition. Mandibular prognathism (“underbite”) was diagnosed by combined extraoral and intraoral examination. Diagnostic features included an elongated and anteriorly positioned mandible, a concave facial profile, nasal depression, failure of lip closure and the lower incisors overriding the upper incisors. Animals lacking these signs were classified as controls (normal occlusion). Population screen. A regional survey of 959 Dolang sheep established a local prevalence of 10.3% for mandibular prognathism. Imaging and histology subset. A subset of 10 cases and 10 controls underwent lateral head radiography, and mandibular bone from these animals was processed for paraffin histology. Hi-C subset (chromatin conformation). Two affected animals (IDs: dibaotian1 and dibaotian2) and two control animals (IDs: duizhao1 and duizhao2) were selected from the Dolang population for deep in situ Hi-C profiling of mandibular bone (Figure 1).

Genomics cohort (blood DNA). Based on this screen, we initiated a broader case–control resource of Dolang sheep for multi-omics studies of mandibular prognathism. In this resource, 200 affected and 200 unaffected animals were clinically examined and blood samples were collected to establish a long-term repository for population-genetic and transcriptomic analyses. Population-level analyses in this 200-case/200-control resource (e.g., selection scans and RNA-based profiling) are part of our broader research program and are not reported in the present pilot in situ Hi-C study.

GWAS cohort (reported elsewhere). In a related case–control GWAS study from our group, blood samples were collected from 175 Dolang sheep (145 affected individuals with mandibular prognathism and 30 phenotypically normal controls). All animals in that GWAS cohort underwent standardized radiographic examination and quantitative morphometric assessment, followed by genome-wide SNP genotyping and association analyses. The GWAS results are presented in a separate manuscript (Cao et al., in press) and are not re-analyzed here, in order to avoid redundancy and duplicate publication.

### 2.3. Study Design and Sample Size Rationale

This study was embedded in a larger case–control effort on Dolang sheep established to investigate mandibular prognathism. Based on a regional survey of 959 Dolang sheep, we estimated a local prevalence of 10.3% for mandibular prognathism in a commercial flock from Makit (Maigaiti) County. From this population, we assembled a case–control cohort of 200 affected and 200 unaffected animals (blood DNA) as a long-term resource for population-genetic analyses (e.g., selection scans, genome-wide association studies and homozygosity mapping) and transcriptomic profiling. Genome-wide SNP genotyping and a case–control GWAS have already been carried out in a related cohort of 145 affected and 30 control Dolang sheep from the same farm, and those GWAS results are reported separately (Hu et al., in press).

As an exploratory pilot investigation of three-dimensional (3D) genome architecture, we performed in situ Hi-C on mandibular bone from a subset of two affected (UNDER group: dibaotian1, dibaotian2) and two control animals (CTRL group: duizhao1, duizhao2). The primary goal of this Hi-C component was to establish the feasibility of 3D genome profiling in ovine mandibular tissue and to generate hypothesis-driven candidate regions, rather than to deliver definitive, population-level estimates of effect sizes. To partially offset the small number of biological replicates, each Hi-C library was sequenced to high depth, targeting approximately 100× nominal Hi-C coverage per animal, which is sufficient to robustly detect A/B compartments, TADs and insulation boundaries in mammalian genomes. Unless specified otherwise, group comparisons are reported with effect sizes and false discovery rate (FDR) control, and all Hi-C findings are interpreted as discovery-oriented signals that require validation in larger cohorts and with additional data types. The present manuscript is therefore restricted to the pilot in situ Hi-C analysis, whereas population-genetic and transcriptomic analyses in the broader cohorts are or will be reported separately.

### 2.4. Tissue Collection and Crosslinking

Animals were clinically examined on farm and classified as affected (mandibular prognathism) or unaffected (normal occlusion) based on standardized extraoral and intraoral criteria. For Hi-C and histology, mandibular bone samples were obtained under veterinary supervision. Animals were humanely handled in accordance with farm management and ethical guidelines; mandibular bone (~100–150 mg) with adjacent soft tissue was collected post-mortem at routine slaughter, and no animals were euthanized solely for research purposes.

Immediately after collection, specimens were rinsed in ice-cold phosphate-buffered saline (PBS), finely minced and transferred to 1% methanol-free formaldehyde for 10 min at room temperature to crosslink chromatin. Crosslinking was quenched with 0.125 M glycine for 5 min, followed by two PBS washes. The pelleted material was then snap-frozen in liquid nitrogen and stored at −80 °C until Hi-C library preparation.

### 2.5. In Situ Hi-C Library Preparation and Sequencing

Libraries were prepared following the standard in situ Hi-C workflow described by Rao et al. [17], with minor adaptations for sheep mandibular tissue. Briefly, permeabilized nuclei were digested with MboI at 37 °C, 5′ overhangs were filled in with biotin-dATP and proximity ligation was performed under dilute conditions. After reversal of crosslinks, DNA was purified using AMPure-type paramagnetic beads (Beckman Coulter, Brea, CA, USA), and biotinylated junctions were captured on streptavidin beads. Libraries were then end-repaired, A-tailed, ligated to indexed adapters and PCR-amplified, with cycle numbers determined by qPCR pre-tests. Library quality was assessed by capillary electrophoresis (with enrichment of ~300–700 bp fragments) and junction-specific qPCR. Indexed libraries were pooled and sequenced on a DNBSEQ-T7 instrument (MGI Tech Co., Ltd., Shenzhen, China)) to generate paired-end 150 bp reads, targeting ~100× nominal Hi-C depth per animal.

### 2.6. Hi-C Processing with HiC-Pro

Raw FASTQ files were processed with HiC-Pro (v3.1.0) using the in situ pipeline configured for the sheep reference genome and the MboI restriction map (recognition site GATC) [18]. Adapters and low-quality bases were trimmed with fastp (v1.0.1), and read quality was summarized with FastQC (v0.12.1) [19]. Clean reads were aligned to the *Ovis aries* reference genome (GCA_016772045.2) using Bowtie2 (v2.5.4) in end-to-end, very-sensitive mode (—very-sensitive-L 30-N 0) to accommodate chimeric Hi-C ligation products [20]. HiC-Pro removed unmapped, secondary and supplementary alignments, marked and filtered PCR duplicates, assigned reads to restriction fragments and classified read pairs; only pairs mapping to autosomes or sex chromosomes with MAPQ ≥ 30 were retained as valid pairs. For each sample, raw and ICE-normalized contact matrices were generated at multiple resolutions (10 kb, 25 kb, 40 kb, 100 kb, 200 kb, 500 kb and 1 Mb) and balanced by iterative correction (ICE).

### 2.7. Conversion to HiCExplorer Format and Normalization

HiC-Pro contact matrices were converted for downstream analysis with HiCExplorer (v3.7.6) using hicConvertFormat (—inputFormat hicpro —outputFormat h5), supplying the per-resolution .matrix and .bed files generated by HiC-Pro [21]. Converted files were inspected with hicInfo (v 3.7.2). To harmonize normalization across tools, matrices were ICE-balanced (either inherited from HiC-Pro or re-balanced with hicCorrectMatrix), with filter thresholds chosen from matrix-value distributions (typically—filterThreshold-1.5 5). Coverage comparability and sparsity were assessed using distance–decay curves (hicPlotDistVsCounts) and standard matrix summaries prior to TAD calling and locus-level visualization.

### 2.8. Replicate Concordance and Group Merging

Biological replicate concordance was evaluated at intermediate resolutions (50 kb and 100 kb) using hicCorrelate (v 3.7.2) (Pearson and Spearman coefficients) and distance-stratified correlations. Replicates with within-group correlation ≥ 0.80 were deemed concordant and were merged at the group level for descriptive visualization with hicSumMatrices. All inferential analyses (boundary detection and between-group comparisons) were performed on individual replicates to preserve biological variance.

### 2.9. TAD and Boundary Identification

Topologically associating domains (TADs) were identified with hicFindTADs (v 3.7.2) on ICE-balanced contact maps at fine (25–40 kb) and intermediate (50–200 kb) resolutions, using parameters recommended for mammalian genomes (—minDepth 250, —maxDepth 2,000,000—step 25,000) and controlling for multiple testing with Benjamini–Hochberg FDR (—correctForMultipleTesting fdr). The insulation-based delta and TAD-separation score tracks were used to define boundary positions and strengths, and outputs were exported as BED/BEDGRAPH files for downstream overlap, proximity and density analyses. Robustness was assessed by confirming broadly similar TAD architectures across resolutions and by verifying boundary reproducibility across biological replicates.

### 2.10. Group-Wise Comparison of TAD Architecture

Per-sample boundary calls (BED) at the analysis resolutions (typically 40 kb and 100–200 kb) were first harmonized by merging nearby peaks within ±1 bin using bedtools (v2.26.0; sort, merge) to produce non-redundant boundary sets for each sample [22]. Within each group (CTRL vs. UNDER), consensus boundaries were then derived under two stringencies: a lenient set (present in ≥1 replicate) and a stringent set (present in both replicates within ±1 bin). Between-group status was classified as conserved (overlap within ±1 bin and Jaccard ≥ 0.50 on the union set) or gain/loss (present in ≥1 UNDER replicate and absent from both CTRL replicates, or the converse).

For quantitative effects, boundary-strength (BS) tracks from hicFindTADs (BEDGRAPH) were used to compute ΔBS = BS_UNDER − BS_CTRL at nearest boundaries paired within ±50 kb (bedtools closest). Pairings without a partner within this window were excluded from ΔBS testing but retained for qualitative gain/loss tallies. To account for replicate structure, linear mixed-effects models were fitted in R (v4.5.1; R Foundation for Statistical Computing, Vienna, Austria) using the lme4 package (v1.1-14), with group (UNDER vs. CTRL) as a fixed effect and animal ID as a random intercept; sex and age were included as covariates when recorded. Wald *p*-values were adjusted by the Benjamini–Hochberg procedure, and unless otherwise noted, discoveries were reported at FDR < 0.10. In parallel, 1 Mb boundary-density windows were summarized (counts per 1 Mb using bedtools map) to highlight focal hotspots (Δ = UNDER − CTRL), which were then used for locus-level visualization.

### 2.11. Chromatin Loop Detection and Visualization

Where sequencing depth permitted, chromatin loops were called on 10–25 kb ICE-balanced matrices using hicDetectLoops (v 3.7.2) with default FDR control. Loops observed within ±1 bin in both replicates of a group were considered consensus loops. For descriptive comparison, loop enrichments were summarized from observed/expected scores and juxtaposed with TAD architectures at representative loci. Genome-wide contact patterns, TADs, boundaries and loops were visualized using hicPlotMatrix (v 3.7.2), hicPlotTADs (v 3.7.2) and hicPlotProfile (v 3.7.2). Locus-level panels included CTRL versus UNDER heatmaps, boundary overlays, 1 Mb boundary-density bars and gene tracks to facilitate biological interpretation.

### 2.12. Gene Annotation of Differential Regions

Group-wise boundary sets were built at 40 kb resolution by merging per-sample calls within ±1 bin to obtain non-redundant CTRL and UNDER BED files. The genome was tiled into 1 Mb windows, boundary counts were computed per group and Δ = UNDER − CTRL was derived; windows with Δ ≥ 2 were taken as UNDER-enriched hotspots. All differential intervals (hotspots and group-specific gains/losses) were de-duplicated per chromosome using a ±40 kb merge to yield the final region list. Gene features were parsed from the study genomic annotation file to generate gene bodies, transcription start sites (TSS; strand-aware, 1 bp) and promoters (TSS ± 2 kb). For each region, we reported (i) promoter overlap, (ii) gene-body overlap and (iii) the nearest TSS (within ±1 Mb, with distance recorded). When multiple criteria applied, priority was assigned in the order promoter > gene body > nearest TSS. Candidate genes were ranked for display as Tier 1 (promoter), Tier 2 (gene body) or Tier 3 (nearest TSS within ≤250 kb). The annotated tables (region class, coordinates, overlaps, nearest gene/distance and tier) are provided in Table 1 and Supplementary Table S1 and were used to prioritize candidate genes at each differential region.

## 3. Results

### 3.1. Sequencing Output and Hi-C Mapping Quality

DNBSEQ-T7 (PE150) Hi-C libraries yielded a median of 463.1 million valid (de-duplicated) Hi-C pairs per sample (range 439.1–532.2 million). Across samples, the cis/total contact fraction averaged 54.86% (range 52.37–59.97%) (Table 2). Within cis contacts, short-range interactions (as reported by HiC-Pro) accounted for a mean of 9.8% of cis pairs (range 9.0–11.3%), with the remainder being long-range. The duplicate rate within valid interactions averaged 5.89% (range 4.67–8.45%). Overall, these metrics are consistent with high-quality proximity ligation suitable for downstream compartment, TAD and boundary analyses.

### 3.2. Reproducibility Across Biological Replicates

At 50 kb resolution, within-group replicate correlations were high, and similar patterns were observed at 100 kb. Given replicate concordance ≥ 0.80, replicate-merged maps were generated at the group level for visualization only, whereas all statistical inferences were based on individual replicates.

### 3.3. Genome-Wide TAD Architecture

**Figure 2:**
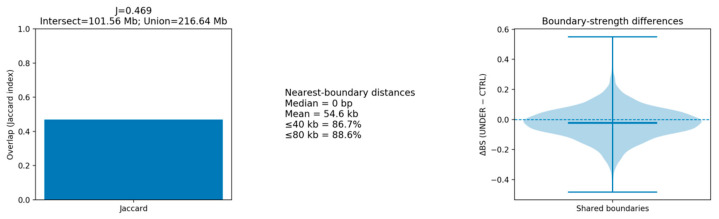
Global concordance of TAD boundaries between CTRL and UNDER groups in 40 kb resolution. The Jaccard index is J = 0.469 (Intersect = 101.56 Mb; Union = 216.64 Mb), indicating that nearly half of all boundaries are shared. Nearest-boundary distances have a median of 0 bp and a mean of 54.6 kb; 86.7% of boundaries fall within 40 kb and 88.6% within 80 kb of a counterpart, showing that most boundaries nearly coincide between groups.

Using hicFindTADs on ICE-balanced contact maps at 40 kb resolution, we identified 2769–2790 TADs per sample (median domain size ~0.70 Mb, with per-sample medians ranging from 0.68 to 0.72 Mb; IQR 0.48–1.04 Mb; Table 3). TAD architectures were broadly consistent across nearby resolutions (Figure 2 and Figure S1A,B). The distributions of boundary strength (insulation delta) overlapped substantially between groups, with group-level medians BS_CTRL = −0.022 and BS_UNDER = −0.016, indicating a largely conserved genome-wide domain framework with focal differences explored in subsequent sections.

### 3.4. Boundary Conservation Between Groups at 40 kb

At a working resolution of 40 kb, we merged replicate-specific boundaries within each group (merge distance = one bin) and quantified cross-group concordance. The resulting group-merged boundary sets showed moderate conservation, with a Jaccard index of 0.469 derived from an intersection of 101.56 Mb and a union of 216.64 Mb (n_intersections = 2341), indicating that roughly half of the boundary landscape is shared between underbite (UNDER) and control (CTRL) animals and that large-scale chromatin partitioning is broadly similar between phenotypes.

To complement this overlap metric with a quantitative insulation comparison, we paired n = 5312 boundaries supported in both groups by nearest midpoints and computed ΔBS = BS_UNDER − BS_CTRL at each boundary. The ΔBS distribution was centered near zero (median −0.0195; IQR −0.0850 to 0.0461; median |ΔBS| = 0.0672), and after Benjamini–Hochberg correction no boundary reached FDR < 0.10. Together, the overlap and ΔBS results argue against genome-wide reorganization of the TAD boundary landscape at 40 kb under the current design, while allowing for subtle, localized differences at specific loci. Consistently, a pilot analysis at 500 kb resolution likewise did not reveal extensive insulation shifts, supporting a model of overall boundary conservation with focal remodeling rather than global architectural change.

### 3.5. Boundary Alignment Is Dominated by Coincident Loci and Sub-Bin Shifts

At 40 kb resolution, nearest-boundary distances clarified the nature of cross-group differences. The median offset was 0 bp and the mean offset was 54.6 kb, with 86.7% and 88.6% of boundaries aligning within ≤40 kb and ≤80 kb, respectively. Because one 40 kb bin matches the calling granularity, these metrics indicate that most boundaries are coincident or shifted by only one to two bins, representing micro-relocations rather than large positional changes. This pattern explains how the Jaccard overlap can fall below 0.5 while nearest-neighbor distances still center tightly at zero: small shifts reduce strict set overlap without implying wholesale boundary movement.

### 3.6. Global Conservation with a Slight Net Reduction in Boundaries in UNDER

Allowing a ±40 kb tolerance to accommodate minor shifts, we counted 453 CTRL-specific and 362 UNDER-specific boundaries, suggesting a slight net reduction in boundaries in UNDER. Consistently, boundary-wise insulation differences across n = 5312 shared boundaries were centered near zero (median ΔBS = −0.0195; IQR −0.0850 to 0.0461; median |ΔBS| = 0.0672), and no boundary remained significant after Benjamini–Hochberg correction (FDR < 0.10), arguing against genome-wide changes in insulation strength.

### 3.7. Discrete Remodeling Loci Nominated by 1 Mb Boundary-Density Hotspots 

**Figure 3:**
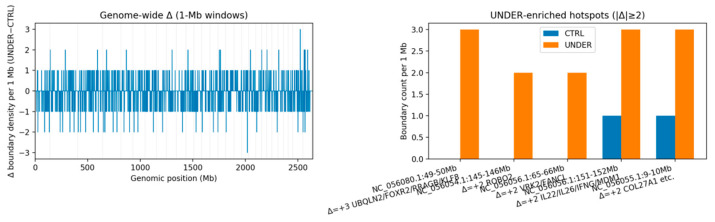
Genome-wide changes in TAD-boundary density between UNDER and CTRL. (**Left**)**:** 1 Mb windows across all chromosomes. Most windows show small or no differences (|Δ| < 2), whereas a limited number of hotspots with |Δ| ≥ 2 (highlighted in orange) stand out from the background. (**Right**)**:** 1 Mb window at selected UNDER-enriched hotspots. In each case, UNDER animals (orange) show more boundaries than CTRL animals (blue), nominating discrete regions near craniofacial-relevant genes (e.g., *ROBO2*, *COL27A1*, *VRK2*, *IL22/IL26/IFNG*) for more detailed inspection.

**Figure 4:**
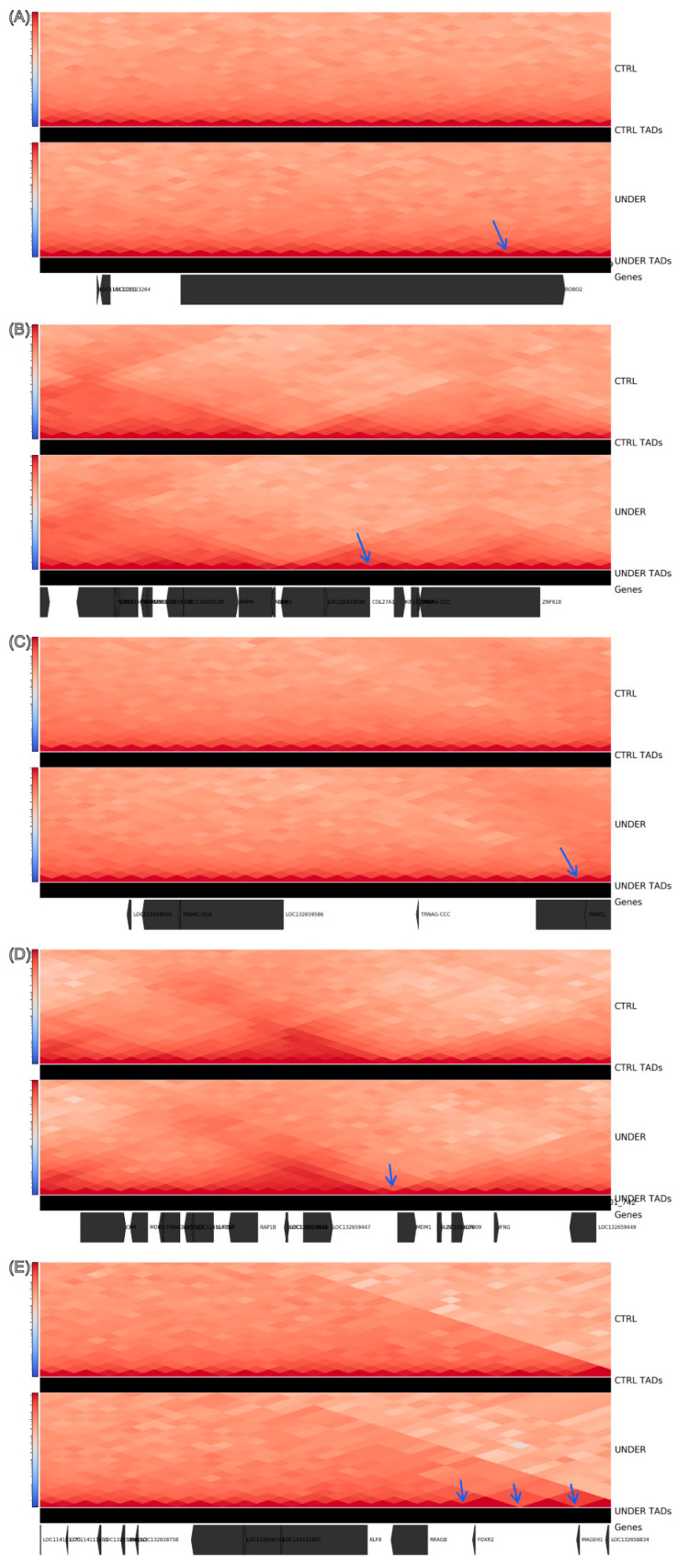
Representative hotspots showing localized remodeling of 3D genome organization in UNDER vs. CTRL animals. For each 1 Mb locus (**A**–**E**), the upper heatmaps show group-merged Hi-C contact maps at 40 kb resolution for CTRL and UNDER, and the tracks immediately below show the corresponding TAD calls. The bottom track shows the annotated genes in each region. Arrows mark TAD boundaries whose boundary strength is higher in UNDER than in CTRL. (**A**) Hotspot near *ROBO2*: a sharper boundary in UNDER (arrow) subdivides a larger CTRL domain and increases insulation close to *ROBO2*. (**B**) Hotspot near *COL27A1*: a stronger boundary in UNDER (arrow) splits the CTRL domain and delimits a smaller TAD containing *COL27A1*. (**C**) Hotspot near *VRK2*/FANCL: a strengthened boundary in UNDER (arrow) sharpens local insulation and partly isolates *VRK2* from its upstream neighborhood. (**D**) Hotspot near the *IL22*/*IL26*/*IFNG*–*MDM1* cytokine cluster: a stronger flanking boundary in UNDER (arrow) enhances insulation around this immune-related gene cluster. (**E**) Hotspot near FOXR2–MAGEH1: stronger boundaries in UNDER (arrows) segment the contact domain more strongly than in CTRL, indicating increased insulation flanking FOXR2 and MAGEH1.

To localize remodeling, we profiled boundary density in 1 Mb sliding windows and contrasted groups (Δ = UNDER − CTRL) (Figure 3). Using a stringent threshold of |Δ| ≥ 2 boundaries/Mb, we detected 18 UNDER-enriched windows (17 overlapping annotated genes) and 38 CTRL-enriched windows (Table 3; Table S1). Although CTRL had more enriched windows overall—consistent with its larger set of group-specific boundaries—UNDER showed several salient, gene-proximal hotspots, including (Figure 4):

NC_056080.1: 49–50 Mb (Δ = +3; CTRL = 0 vs. UNDER = 3), overlapping UBQLN2, FOXR2, MAGEH1, RRAGB and KLF8;

NC_056054.1: 145–146 Mb (Δ = +2), overlapping *ROBO2* (a guidance receptor family member with established roles in craniofacial development);

NC_056056.1: 65–66 Mb (Δ = +2), overlapping *VRK2* and FANCL;

NC_056056.1: 151–152 Mb (Δ = +2), overlapping the *IL22*/*IL26*/*IFNG* cytokine cluster and *MDM1*;

NC_056055.1: 9–10 Mb (Δ = +2), overlapping AKNA, KIF12, AMBP, WHRN, ZNF618 and *COL27A1*, which encodes a collagen linked to skeletal and cartilage processes.

At these loci, UNDER maps exhibited additional and/or stronger boundaries relative to CTRL, effectively subdividing local domains and implying increased local insulation rather than large-scale domain shifts.

### 3.8. Sensitivity, Robustness, and Interpretation

Three complementary readouts—(i) global set similarity (Jaccard ≈ 0.47), (ii) positional concordance (nearest-boundary median = 0 bp, 86–89% within ≤1–2 bins) and (iii) windowed density contrasts (stringent |Δ| ≥ 2 hotspots)—paint a coherent picture: there is no genome-wide overhaul of TADs or boundaries, but rather boundary micro-shifts with focal density gains in UNDER at biologically relevant loci. The stringent hotspot cutoff ensures high specificity; relaxing the threshold to Δ ≥ 1 would increase sensitivity, and we report the corresponding results as a robustness check without altering the qualitative conclusions (Supplementary Table S1). Collectively, these results support a model of broad architectural conservation with localized remodeling at candidate craniofacial neighborhoods (e.g., *ROBO2*, *COL27A1*, VRK2), which are prioritized for follow-up by RNA-seq (differential expression and isoforms), allelic-imbalance analyses or variant-enrichment tests within ±0.5–1 Mb.

## 4. Discussion

### 4.1. Local Boundary Gains on a Globally Conserved Scaffold

From a technical standpoint, the choice of in situ Hi-C was directly aligned with our main hypothesis. We sought to determine whether mandibular prognathism in Dolang sheep is accompanied by a wholesale reorganization of TAD architecture or, alternatively, by focal changes in boundary strength and density at specific craniofacial loci. Genome-wide in situ Hi-C at 40 kb resolution provides quantitative maps of A/B compartments, TADs and insulation boundaries across the entire ovine genome in mandibular bone, which allowed us to test this hypothesis at the megabase scale without restricting the analysis to pre-selected loci [15,17,23].

Despite the underbite phenotype, the megabase-scale TAD topology is largely preserved between groups. A moderate cross-group Jaccard index, together with nearest-boundary medians at 0 bp, indicates that most differences reflect sub-bin to one-bin offsets and modest changes in boundary strength rather than wholesale repositioning of domains [24]. In other words, the majority of boundaries remain coincident or nearly so, with only minor micro-shifts in their genomic coordinates. This pattern is consistent with the broader literature, in which TAD frameworks are generally stable across individuals and conditions, whereas trait- or stimulus-associated remodeling more often manifests as boundary sharpening or weakening and sub-TAD partitioning that fine-tunes local regulatory neighborhoods without altering the global scaffold [25].

Mandibular prognathism in humans and livestock has emerged as a genetically complex and moderately to highly heritable trait, with multiple loci and pathways implicated across recent genome-wide and candidate-gene studies [1,26,27]. Within this context, our pilot Hi-C analysis does not attempt to nominate new causal variants directly, but instead asks whether the three-dimensional regulatory architecture around such loci is measurably altered in animals with extreme underbite [17,28].

The boundary-density screen converges on a small set of UNDER-enriched loci where insulation increases (|Δ| ≥ 2 per 1 Mb window), and a coherent functional picture emerges when these structural signals are read in their local genomic context. At each hotspot, the magnitude and direction of the change—net gains of +2 to +3 boundaries—are consistent with domain subdivision and sharpening of insulation, a configuration expected to restrict enhancer traffic across newly reinforced borders while preserving the long-range TAD scaffold [29,30]. This architectural motif aligns with the gene content in these intervals. Similar patterns of local domain refinement at otherwise conserved scaffolds have been described at developmental loci in limb and craniofacial models, where modest changes in insulation are sufficient to bias enhancer-promoter communication and alter morphology. In Dolang sheep, the UNDER-enriched hotspots we observe may therefore represent analogous “regulatory bottlenecks” at craniofacial genes, translating subtle 3D architectural shifts into measurable mandibular phenotypes.

For example, *ROBO2* resides within a window exhibiting Δ = +2; strengthened insulation here would be predicted to narrow the promoter’s sphere of regulatory influence, biasing it toward within-domain enhancers and away from cross-boundary contacts, which offers a plausible route to dosage tuning during craniofacial growth [31]. Likewise, the *COL27A1*-containing interval (Δ = +2) lies in a collagen-rich regulatory neighborhood relevant to cartilage and mandibular development [32]. Additional boundaries in this region would be expected to restrict or re-route enhancer–promoter pairing, shifting transcriptional output without necessitating compartment switches. The *VRK2*/FANCL window suggests a similar principle for a developmental kinase locus [33], whereas increased insulation across the *IL22*/*IL26*/*IFNG*–*MDM1* region raises the possibility that immune- or cilium/centrosome-linked pathways intersect mandibular morphogenesis via stromal or osteogenic cell states present in bone tissue [34].

Mechanistically, such boundary gains could arise from increased usage of CTCF motifs, orientation-dependent loop-extrusion stalling or chromatin-state sharpening at edge elements; any of these mechanisms would deepen the local insulation troughs visible on the Hi-C maps [35]. Importantly, the hotspot pattern helps to reconcile our global metrics [36]. The moderate Jaccard index, despite nearest-boundary medians at 0 bp, implies that strict set overlap can fall even when the functional scaffold changes only subtly, because boundaries that are micro-shifted or newly strengthened are treated as distinct. At the same time, ΔBS medians near zero indicate that the large majority of shared boundaries retain comparable strength, with remodeling confined to selected neighborhoods where density and strength jointly increase [37,38].

If insulation is indeed strengthened at these loci, we expect mandibular RNA-seq to reveal cis-proximal expression changes (including possible isoform shifts) for the hotspot genes or their immediate neighbors, and CTCF/cohesin profiling (ChIP-seq or CUT&Tag) to show elevated occupancy and convergent motif organization at reinforced borders [39,40]. Variant analyses in the broader case–control cohort could then test whether regulatory alleles are enriched within ±0.5–1 Mb of remodeled edges, particularly near candidate enhancers and loop anchors. Together, these focal boundary gains provide a mechanistic layer that complements existing genetic and morphometric studies of mandibular prognathism, shifting the emphasis from where susceptibility loci are located to how regulatory domains around those loci may be wired and insulated in animals with underbite [25,31,32,33,34].

Alternative interpretations remain possible. For example, differences in cell-state composition within mandibular bone (e.g., osteoblast, chondrocyte, stromal and immune compartments) could modulate apparent insulation if boundary usage differs across cell types [41]. Such confounding, however, would be expected to affect many regions broadly, which we do not observe. Instead, the tight localization of boundary gains and their alignment with biologically pertinent genes argue for a mechanistic link between boundary strengthening and mandible-related regulation. Practically, this prioritizes the *ROBO2*, *COL27A1*, *VRK2* and cytokine-cluster windows for integrated follow-up—combining chromatin profiling, allele-aware expression and targeted fine-mapping—to determine whether boundary remodeling is primary (sequence encoded) or secondary (state dependent), and to quantify its contribution to the underbite phenotype.

### 4.2. Practical Implications and Testable Predictions

The data therefore support a model of localized boundary strengthening superimposed on a conserved global scaffold [41]. This conclusion follows directly from the in situ Hi-C maps: global distributions of TAD size and boundary strength are highly similar between groups, yet the same maps reveal discrete 1 Mb windows with increased boundary density and insulation in UNDER at craniofacial loci such as ROBO2, COL27A1 and VRK2.

This model yields several concrete, testable predictions. First, refined Hi-C analyses should confirm higher local insulation in UNDER at the nominated loci, reflected in stronger insulation scores/ΔBS, sharper insulation troughs and more robust boundary calls [42]. Second, mandibular transcriptomes should show cis-proximal transcriptional effects within the affected domains—for example, differential expression and/or isoform shifts for *ROBO2*, *COL27A1*, *VRK2* and nearby genes—rather than diffuse trans effects. Third, the regulatory architecture at strengthened edges should be consistent with reinforced borders, including enriched and convergently oriented CTCF motifs, increased CTCF/cohesin occupancy and accessible chromatin at boundary elements [43].

These predictions are directly addressable within our ongoing research program, which combines Hi-C insulation and boundary calling, RNA-seq differential expression/isoform/allelic-imbalance analyses, and, where feasible, CTCF/cohesin CUT&Tag/ChIP-seq or ATAC-seq within ±0.5–1 Mb of the hotspots [44]. By design, the present study is exploratory and hypothesis-generating, but it yields a focused set of loci and molecular readouts that can be evaluated in the larger 200 cases/200 controls resource.

### 4.3. Limitations

This pilot in situ Hi-C study includes few animals per group and emphasizes five high-confidence 1 Mb hotspots, which limits power to detect subtler or cell-type–restricted effects at finer resolutions [45]. Tissue heterogeneity in mandibular bone (osteoblast, chondrocyte, stromal and immune compartments) and choices of bin size, normalization and boundary-calling parameters may also influence boundary detection and ΔBS estimates. Although gene proximity at the highlighted windows is compelling, causality cannot be inferred from contact maps alone; independent expression (RNA-seq), chromatin (CTCF/cohesin, ATAC/CUT&Tag) and variant data are required to triangulate mechanisms [36]. Technical factors—including reference assembly and annotation completeness, restriction/ligation biases and cross-sample matrix balancing—could further introduce modest uncertainty.

At the same time, the in situ Hi-C approach used here was well matched to our primary question: it provided genome-wide, quantitative maps of TADs and insulation boundaries in mandibular bone, allowing us to distinguish a largely conserved megabase-scale scaffold from localized gains in boundary density at specific craniofacial loci. In future work, higher-resolution or targeted 3D genomics methods such as capture Hi-C, HiChIP or Micro-C will be valuable to refine enhancer–promoter wiring within these intervals, but they build on—and do not replace—the megabase-scale view provided by in situ Hi-C in this pilot study [23,46].

Despite these constraints, the convergence of multiple metrics (boundary overlap, nearest-boundary distance, ΔBS and density hotspots) and the biological plausibility of several craniofacial-relevant loci provide a coherent framework and clear priorities for scaled validation [42]. We therefore view the present work as a first, pilot application of in situ Hi-C to mandibular prognathism in a livestock species, laying the groundwork for mechanistic dissection of three-dimensional genome organization at mandibular prognathism–relevant loci in Dolang sheep and beyond.

## 5. Conclusions

In this study, we combined clinical phenotyping, imaging, histology and pilot Hi-C profiling to investigate mandibular prognathism in Dolang sheep. We documented a local prevalence of 10.3% in a commercial flock from Makit (Maigaiti) County and established standardized criteria for diagnosing the defect. Deep in situ Hi-C analysis of mandibular bone from two affected and two control sheep revealed that the genome-wide TAD and boundary landscape is broadly conserved between groups, consistent with a stable global 3D genomic scaffold.

Within this conserved framework, we detected a small set of 1 Mb windows where affected animals showed increased TAD-boundary density and strengthened insulation, including regions near *ROBO2*, *COL27A1*, *VRK2*/FANCL and an *IL22*/*IL26*/*IFNG*–*MDM1* cytokine cluster. These focal gains in insulation are consistent with local subdivision of regulatory neighborhoods at craniofacial-relevant loci and suggest a potential contribution of 3D genome remodeling to mandibular prognathism in Dolang sheep.

Because the Hi-C sample size is small and matched whole-genome and transcriptomic data are not yet integrated, our findings should be interpreted as preliminary and hypothesis-generating. Nevertheless, the hotspots identified here provide concrete candidates for follow-up analyses in the existing 200/200 case–control cohort using whole-genome sequencing, genome-wide association, fine-mapping and RNA-seq. More broadly, this work establishes a framework for combining 3D genome architecture with population genetics and functional genomics to study craniofacial traits in livestock and highlights Dolang sheep as a useful large-animal model for mandibular prognathism.

## Figures and Tables

**Table 1:** Representative 1 Mb genomic windows with higher TAD-boundary density in UNDER vs. CTRL (Δ ≥ 2), showing per-group boundary counts and nearby genes. The full list of differential windows is provided in Table S1.

Chr	Start	End	Count_CTRL	Count_UNDER	Delta	Genes
NC_056080.1	49,000,000	50,000,000	0	3	3	UBQLN2/FOXR2/RRAGB/KLF8
NC_056056.1	151,000,000	152,000,000	1	3	2	*IL22*/*IL26*/*IFNG*, *MDM1*
NC_056056.1	65,000,000	66,000,000	0	2	2	*VRK2*/FANCL
NC_056054.1	145,000,000	146,000,000	0	2	2	*ROBO2*
NC_056055.1	9,000,000	10,000,000	1	3	2	*COL27A1* (+AKNA/KIF12/AMBP/WHRN/ZNF618)

**Table 2:** Hi-C contact statistics for four samples.

Sample	Valid_Pairs	Cis	Trans	Cis_Ratio_pct
dibaotian1	444,574,329	234,569,028	210,005,301	52.76
dibaotian2	481,591,477	252,188,788	229,402,689	52.37
duizhao1	439,112,437	238,565,417	200,547,020	54.33
duizhao2	532,221,884	319,149,103	213,072,781	59.97

**Table 3:** TAD summary at 40 kb resolution for four samples.

Sample	Resolution_bp	TAD_count	Median_size_Mb	IQR_lo_Mb	IQR_hi_Mb	Median_BS
duizhao1	40,000	2769	0.72	0.48	1.04	−0.017
duizhao2	40,000	2790	0.72	0.48	1.04	−0.027
dibaotian1	40,000	2780	0.68	0.48	1.04	−0.018
dibaotian2	40,000	2785	0.68	0.48	1	−0.014

## Data Availability

The data presented in this study are available on request from the corresponding author (Wujun-Liu).

## Supplementary Materials

The following supporting information can be downloaded at: https://www.mdpi.com/article/10.3390/ani16010039/s1, Table S1: Full list of 1-Mb genomic windows with higher TAD-boundary density in UNDER versus CTRL groups (Δ boundary count ≥ 2). For each window, the table reports chromosome, genomic start and end coordinates, the number of TAD boundaries in CTRL and UNDER, the difference in boundary count (Δ = UNDER − CTRL), and nearby annotated genes. Figure S1. (A) Boundary strength (insulation delta) at 40 kb; (B) Boundary strength by group.
