# Supplementary figures and images for: Mandibular Prognathism in Dolang Sheep: Hi-C Evidence for Localized TAD Remodeling at Craniofacial Loci
Source: Animals (Basel). 2025 Dec 23;16(1):39. doi: 10.3390/ani16010039 (PMC12784920; doi:10.3390/ani16010039)

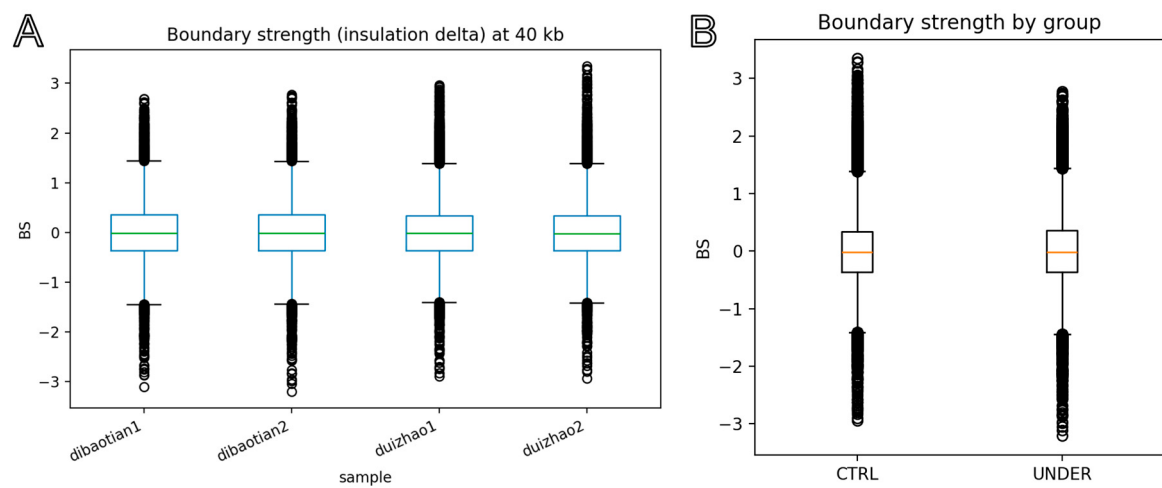

Figure S1. (A) Boundary strength (insulation delta) at 40 kb; (B) Boundary strength by group.

Supplement: Supplementary file 1 [file animals-16-00039-s001.zip › animals-3983905-figure S1.pdf]
